# Unveiling the Potential of Large Language Models in Transforming Chronic Disease Management: Mixed Methods Systematic Review

**DOI:** 10.2196/70535

**Published:** 2025-04-16

**Authors:** Caixia Li, Yina Zhao, Yang Bai, Baoquan Zhao, Yetunde Oluwafunmilayo Tola, Carmen WH Chan, Meifen Zhang, Xia Fu

**Affiliations:** 1 The Department of Nursing The Eighth Affiliated Hospital Sun Yat-sen University Shenzhen China; 2 The School of Nursing Sun Yat-sen University Guangzhou China; 3 The School of Artificial Intelligence Sun Yat-sen University Guangzhou China; 4 The Department of Clinical Research Conestoga College Kitchener, ON Canada; 5 The Nethersole School of Nursing The Chinese University of Hong Kong Hong Kong China

**Keywords:** artificial intelligence, chronic disease, health management, large language model, systematic review

## Abstract

**Background:**

Chronic diseases are a major global health burden, accounting for nearly three-quarters of the deaths worldwide. Large language models (LLMs) are advanced artificial intelligence systems with transformative potential to optimize chronic disease management; however, robust evidence is lacking.

**Objective:**

This review aims to synthesize evidence on the feasibility, opportunities, and challenges of LLMs across the disease management spectrum, from prevention to screening, diagnosis, treatment, and long-term care.

**Methods:**

Following the PRISMA (Preferred Reporting Items for Systematic Reviews and Meta-Analysis) guidelines, 11 databases (Cochrane Central Register of Controlled Trials, CINAHL, Embase, IEEE Xplore, MEDLINE via Ovid, ProQuest Health & Medicine Collection, ScienceDirect, Scopus, Web of Science Core Collection, China National Knowledge Internet, and SinoMed) were searched on April 17, 2024. Intervention and simulation studies that examined LLMs in the management of chronic diseases were included. The methodological quality of the included studies was evaluated using a rating rubric designed for simulation-based research and the risk of bias in nonrandomized studies of interventions tool for quasi-experimental studies. Narrative analysis with descriptive figures was used to synthesize the study findings. Random-effects meta-analyses were conducted to assess the pooled effect estimates of the feasibility of LLMs in chronic disease management.

**Results:**

A total of 20 studies examined general-purpose (n=17) and retrieval-augmented generation-enhanced LLMs (n=3) for the management of chronic diseases, including cancer, cardiovascular diseases, and metabolic disorders. LLMs demonstrated feasibility across the chronic disease management spectrum by generating relevant, comprehensible, and accurate health recommendations (pooled accurate rate 71%, 95% CI 0.59-0.83; *I^2^*=88.32%) with retrieval-augmented generation-enhanced LLMs having higher accuracy rates compared to general-purpose LLMs (odds ratio 2.89, 95% CI 1.83-4.58; *I^2^*=54.45%). LLMs facilitated equitable information access; increased patient awareness regarding ailments, preventive measures, and treatment options; and promoted self-management behaviors in lifestyle modification and symptom coping. Additionally, LLMs facilitate compassionate emotional support, social connections, and health care resources to improve the health outcomes of chronic diseases. However, LLMs face challenges in addressing privacy, language, and cultural issues; undertaking advanced tasks, including diagnosis, medication, and comorbidity management; and generating personalized regimens with real-time adjustments and multiple modalities.

**Conclusions:**

LLMs have demonstrated the potential to transform chronic disease management at the individual, social, and health care levels; however, their direct application in clinical settings is still in its infancy. A multifaceted approach that incorporates robust data security, domain-specific model fine-tuning, multimodal data integration, and wearables is crucial for the evolution of LLMs into invaluable adjuncts for health care professionals to transform chronic disease management.

**Trial Registration:**

PROSPERO CRD42024545412; https://www.crd.york.ac.uk/PROSPERO/view/CRD42024545412

## Introduction

Accounting for nearly three-quarters of deaths worldwide, chronic diseases have become a major challenge to global health [1]. These diseases, primarily cardiovascular diseases, cancers, diabetes, and chronic respiratory diseases, are responsible for 41 million deaths each year globally, 41.5% of which occur in individuals younger than 70 years [1]. Approximately 37.2% of adults worldwide have multiple chronic diseases and experience increased symptom burdens, emergency medical admissions, and health care expenditures [2]. The health burden of chronic diseases is further exacerbated by population aging, urbanization, and unhealthy lifestyles, including a lack of physical activity [3]. Projections indicate that globally, chronic diseases will cause 77.6% of disability-adjusted life years by 2050 [4], and their direct health care costs are expected to reach US $301.8 billion by 2030 [5]. To address this challenge, the World Health Organization 2030 agenda has adopted a global target to reduce premature mortality from chronic diseases by one-third by 2030 [6], highlighting efforts in the prevention, detection, treatment, and long-term management of chronic diseases.

Health care systems for chronic disease management face multidimensional challenges. These systems must process and integrate large volumes of patient data including health records, genomic data, and real-time data (eg, glucose levels) [7,8]. Failure to process such data may lead to fragmented information, impeding the potential for tailored treatment and holistic management of chronic diseases, and ultimately, compromising patient care. Successful chronic disease management also requires day-to-day persistence, with approximately 50% of patients failing to consistently follow prescribed treatment regimens, medications, diets, and physical activities, leading to disease progression and long-term complications [9,10]. Limited access to specialized health care services for chronic diseases presents another challenge, particularly in low-resource health care settings [11]. Approximately 43.3% of people worldwide cannot reach health care facilities within an hour, and those living in rural or remote regions often face increased travel time, costs, and difficulties in accessing health care [12]. This disparity may result in inadequate health promotion, delayed diagnoses, and disrupted treatment of chronic diseases [13]. Collectively, these challenges contribute to suboptimal health outcomes, reinforcing the need for novel approaches to enhance chronic disease management.

Large-language models (LLMs), such as ChatGPT, have emerged as promising solutions for addressing the complexities associated with chronic disease management. These models can be broadly categorized based on their training and application scope: general-purpose LLMs, which are trained to perform a wide range of advanced language tasks, and fine-tuned LLMs, which undergo additional training on specific datasets to specialize in a particular domain [14]. Trained on extensive datasets with billions of parameters, these models are particularly advantageous for analyzing and synthesizing multifaceted health data to assist in developing integrated management plans for chronic diseases [15,16]. For example, LLMs can integrate large-scale clinical notes and laboratory test results to predict the risk of early-stage diabetes before the onset of clinical symptoms, achieving a prediction accuracy score exceeding 0.70 [17]. In addition, LLMs demonstrate proficiency in answering medical questions and providing adaptive communication to patient queries for various chronic diseases, including head and neck cancer [18], gastroesophageal reflux disease [19], and cardiovascular diseases [20]. This could provide patients with personalized health management suggestions, fostering patient engagement and adherence to chronic disease management [21]. Since 2023, LLMs including ChatGPT and Llama have been integrated into real-world electronic health records to support health care professionals in diagnosing diseases and crafting personalized treatment regimens [14]. More importantly, LLMs can be integrated with existing health applications and systems through application programming interfaces to enhance telemedicine [22]. This could enable them to monitor patients’ chronic health conditions, provide diagnostic and treatment information, and aid in follow-up care [23,24], bridging the gap in health care access, especially in low-resource settings [24]. For instance, LLMs have been effectively used to address primary health care concerns and act as essential resources for patients in remote areas [25].

LLMs may introduce a transformative potential to enhance health care practices across the spectrum of chronic disease management. However, several challenges impede their optimal integration in this domain [26,27]. Notably, hallucinations, scenarios in which LLMs generate inaccurate or misleading information, can lead to incorrect diagnoses and inappropriate treatment recommendations [28,29]. However, the transformative force of LLMs necessitates an in-depth understanding of how they can be effectively integrated into current health care systems to enhance chronic disease management. Given the lack of robust evidence in this area, this review was conducted to consolidate the current research findings and provide a comprehensive understanding of the feasibility, opportunities, and challenges associated with the application of LLMs in chronic disease management. These insights can inform future research and practice, and guide the strategic use of LLMs in chronic disease management to alleviate the global burden of chronic diseases.

## Methods

### Review Methodology

A mixed methods systematic review was conducted following the PRISMA (Preferred Reporting Items for Systematic Reviews and Meta-Analyses) statement (Multimedia Appendix 1). The protocol was registered with PROSPERO (CRD42024545412) on May 21, 2024.

### Inclusion and Exclusion Criteria

This review seeks evidence of the potential of LLMs to transform chronic disease management and inform future practices. The detailed inclusion criteria were formulated following the “Population-Intervention-Comparator-Outcomes-Study design” (PICOS) framework [30].

Regarding population, studies conducted among patients with chronic diseases or individuals at a high risk of developing chronic diseases, such as those with obesity, were eligible for inclusion. By contrast, studies that focused on health conditions other than chronic diseases, such as plastic surgery and acute appendicitis, were excluded. Chronic diseases are defined as long-lasting conditions that primarily include cardiovascular diseases, cancer, chronic respiratory diseases, and diabetes [1]. Given that LLMs have not been widely used in clinical settings, studies using simulated patient profiles and scenarios to examine LLMs in chronic disease management were considered eligible.

For interventions, studies were included if they examined LLMs in managing chronic diseases, from prevention to screening, diagnosis, treatment, or follow-up care. LLMs are defined as deep learning models trained on large datasets to comprehend and generate human language text content, which include, but are not limited to, ChatGPT, Bard, BERT, and Llama [14]. Studies focusing on general artificial intelligence, algorithm-based chatbots, and expert systems were excluded.

There were no restrictions on the comparators, including standard comparisons or no comparators.

The study outcomes were the feasibility (eg, accuracy and relevance of responses), opportunities, and challenges (eg privacy issues) of LLMs in managing chronic diseases. This may include the potential benefits of LLMs in enhancing health knowledge, attitudes, and self-care behaviors in chronic disease management.

For study designs, interventions, simulations, and case studies that tested LLMs, including proof-of-concept, feasibility, and experimental studies, were considered eligible. Conference abstracts, commentaries, editorials, and review studies were also excluded.

### Search Strategy

A total of 11 databases, including the Cochrane Central Register of Controlled Trials, CINAHL, Embase, IEEE Xplore, MEDLINE via Ovid, ProQuest Health & Medicine Collection, ScienceDirect, Scopus, Web of Science Core Collection, China National Knowledge Internet, and SinoMed, were searched on April 17, 2024. By conducting an initial search in MEDLINE, 37 search terms (Table S1 in Multimedia Appendix 2) were developed about LLMs and outcomes of interest, including “large language model,” “generative pretrained transformer,” “ChatGPT,” and “self-care.” The titles, abstracts, and subject-heading fields were searched to identify relevant studies. Truncations and Boolean operators were applied to ensure the comprehensive retrieval of relevant literature. Two medical librarians refined the search strategy by reviewing detailed search records on MEDLINE. A manual search of the included studies was performed to identify additional relevant studies. There were no restrictions on publication language, date, or type. Table S2 in Multimedia Appendix 2 presents the complete search strategy for each database.

### Study Screening

The search results were exported to Covidence (Veritas Health Innovation) to eliminate duplicate studies. Two authors (CL and YZ) screened the titles and abstracts, followed by full-text reviews. The exclusion decisions made during the full-text screening were also documented. Discrepancies were resolved through discussions with a third reviewer (XF).

### Data Extraction

Data were extracted using a pilot test and a standardized data extraction form. The form encompassed the study authors, country of origin, study design, chronic health conditions, and characteristics of LLMs, including the name, opportunities, and challenges of LLMs in chronic disease management. One reviewer (CL) independently extracted the data, which were proofread by a second author (YZ) and agreed upon by all authors.

### Data Synthesis

A narrative synthesis was conducted in which the characteristics, feasibility, opportunities, and challenges of LLMs in chronic disease management were described, as reported in the included studies. The synthesis process [31] began with an iterative reading of the study results for familiarization. During this initial process, narrative concepts such as the actionability and readability of LLM responses were identified. Inductive coding was used to capture the essence of these narrative concepts [31]. Codes were subsequently categorized into broader themes (eg, increasing knowledge and awareness) representing the feasibility, opportunities, and challenges of LLMs in chronic disease management. To elucidate and organize the findings, a thematic map was formulated, offering a structural framework for delineating the relationships among the identified themes and key codes (eg, linking health resources). Initial coding was conducted by the first author (CL) and cross-verified by the second author (YZ). All themes were collaboratively refined and a consensus was achieved among all authors.

To synthesize the feasibility outcomes of the LLMs, meta-analyses were conducted using STATA (version 18.0; StataCorp LLC). Pooled accuracy rates, odds ratios comparing accuracy rates of LLMs, and effect sizes for readability scores with 95% CIs were calculated. The statistical significance level was set at *P*<.05. Heterogeneity was assessed using *I^2^* statistics (*I^2^* of 25%, 50%, and 75% indicating low, moderate, and high heterogeneity, respectively) and *Q* statistics (*P*<.10 indicating statistically significant heterogeneity) [32]. Owing to the high heterogeneity among the included studies, the random-effects Dersimonian-Laird model was used in the analyses. A sensitivity analysis was conducted using the leave-one-out approach to evaluate the robustness of the pooled analyses.

### Quality Assessment

A rating rubric was used to assess the quality of the methodology used in simulation-based studies. The rubric contained 16 items: study design, sample size, simulation development and implementation, and study instruments [33]. Each rubric item was graded on a scale of 0-4, and total scores were converted into percentage scores by averaging the total number of appraisal questions eligible for the study [33]. In quasi-experimental studies, the Risk of Bias in Nonrandomized Studies of Interventions tool was used to assess the risk of bias due to confounding factors, participant selection, classification of interventions, deviations from intended interventions, missing data, outcome measurements, and selection of reported outcomes [34]. Each domain and overall methodology were rated as having a low, moderate, serious, or critical risk of bias. Two reviewers (CL and YZ) independently appraised the study quality, and disagreements were resolved through discussion.

## Results

### Search Findings

**Figure 1:**
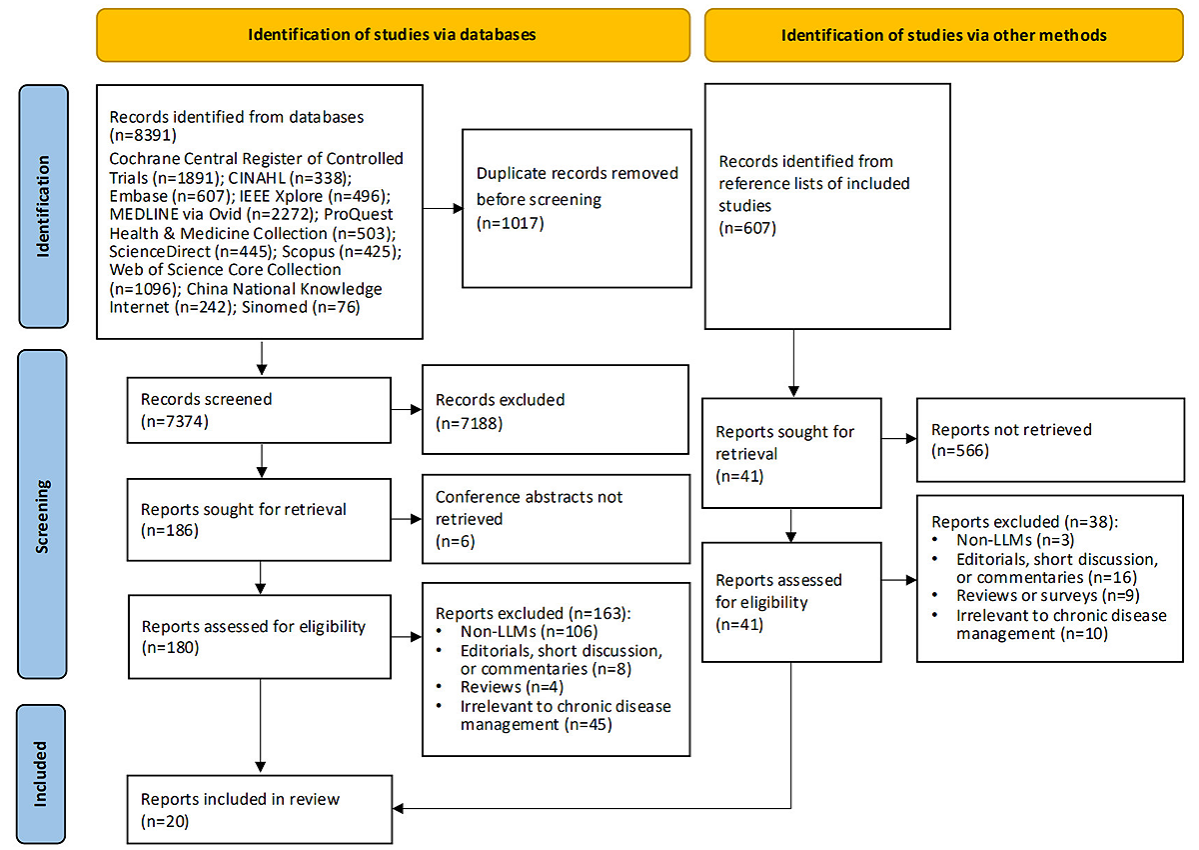
The PRISMA flowchart. LLM: large language model; PRISMA: Preferred Reporting Items for Systematic Reviews and Meta-analysis.

A database search yielded 8391 records. After removing 1017 duplicates, 7374 titles and abstracts remained. Of the 180 full-text studies retrieved, 163 studies were excluded, primarily because of their irrelevance to LLMs or chronic disease management, leaving 17 eligible studies. An additional 607 records were identified by manually searching the reference lists of 17 eligible studies, resulting in the inclusion of 20 studies for this review (Figure 1).

### Study Characteristics

**Figure 2:**
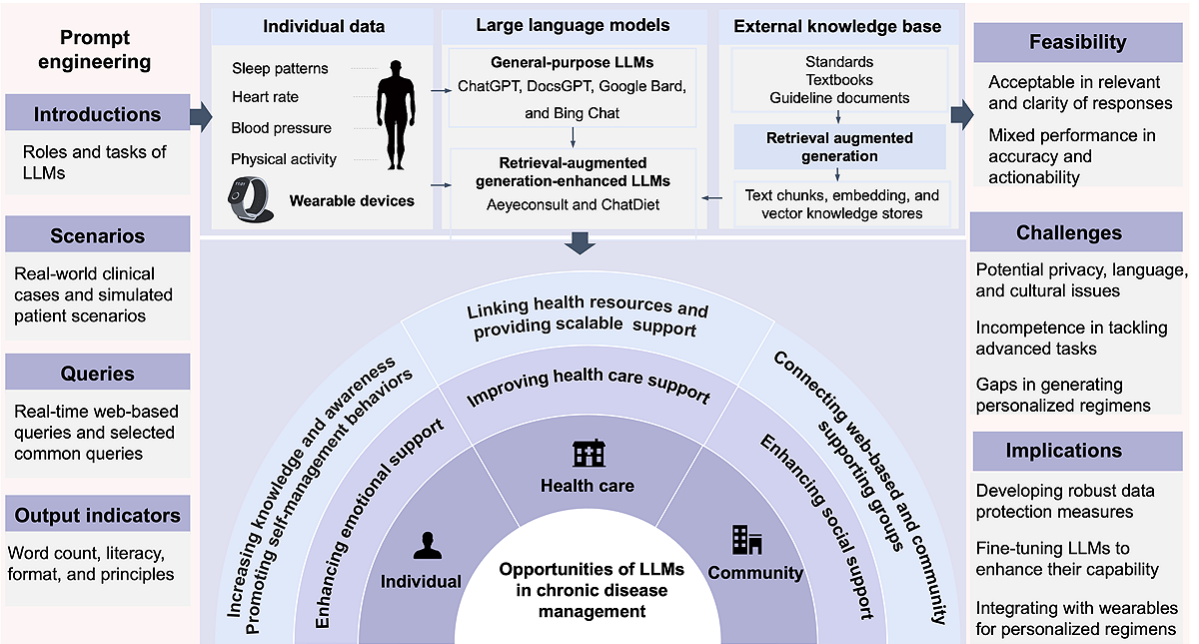
Characteristics, feasibility, opportunities, and challenges of LLMs in chronic disease management. LLM: large language model.

Published between 2023 and 2024, the included studies mainly originated from high-income countries (12/20, 60%), including the United States (n=6) [35-40], Australia (n=2) [41,42], Canada (n=2) [43,44], Singapore (n=1) [45], and South Korea (n=1) [46]. The included studies generally used general-purpose LLMs (17/20, 85%), such as ChatGPT, DocsGPT, Google Bard, and Bing Chat, to manage a spectrum of chronic illnesses, including cancer, cardiovascular diseases, metabolic disorders, respiratory diseases, musculoskeletal disorders, mental health disorders, and substance-use disorders (Table 1). However, direct deployment often lacks the specificity required to manage chronic diseases. Three studies enhanced LLMs through retrieval-augmented generation, which combines LLMs with access to an external knowledge base through a retrieval mechanism [38,39,45]. For example, Lim et al [45] embedded colorectal cancer screening guidelines, decomposed them into manageable textual chunks, and leveraged semantic encoding to facilitate accurate retrieval by ChatGPT 4.0 to generate context-aware screening recommendations. Retrieval-augmented generation also enhances the LLM applicability in managing ophthalmology issues [38] and recommending personalized nutrition regimens [39]. Most studies (15/20, 75%) used simulation-driven or proof-of-concept designs that did not involve human participants (Table 1). Only three quasi-experimental studies involved real-world clinical implementation of LLMs among patients, and their sample sizes ranged from 24 to 72 [21,47,48]. The findings of this review are shown in Table 1 and Figure 2. A detailed overview of the study characteristics is provided in Multimedia Appendix 3 [21,35-53].

**Table 1:** Characteristics of the included studies (n=20).

Author, year, and country	Study design	Chronic diseases	LLM^a^ types	Outcome assessment	Key study findings
AI-Anezi (2024) [47], Saudi Arabia	Quasi-experimental study	Cancer, diabetes, and kidney failure	ChatGPT 3.5, engaged by participants for ≥15 min daily for 2 weeks	Semistructured interviews	ChatGPT 3.5 improved disease awareness, health behaviors, and accessible support while reducing specialist reliance, yet faces issues with disease diagnosis, empathy, data privacy, and managing complex conditions.
Alanezi et al (2024) [48], Saudi Arabia	Quasi-experimental study	Chronic mental health conditions	ChatGPT 3.5, engaged by participants for ≥15 min daily for 2 weeks	Semistructured interviews	ChatGPT 3.5 enhanced mental health literacy and self-care and delivered crisis interventions. It faces challenges in data privacy, accuracy, and catering to cultural and linguistic diversities.
Alanezi (2024) [21], Saudi Arabia	Quasi-experimental study	Cancer	ChatGPT 3.5, engaged by participants for 2 weeks	Focus group interviews	ChatGPT 3.5 improved cancer knowledge, self-management, emotional aid, and social resource access; however, it faces privacy, reliability, and personalization challenges.
Aliyeva et al (2024) [35], United States	Simulation study	Severe hearing loss	ChatGPT 4.0, posed with five postoperative management questions	Survey	ChatGPT 4.0 had 100% accuracy, rapid response times, 98% clarity, and 92% relevance in its recommendations.
Choo et al (2024) [46], South Korea	A simulation study	Colorectal cancer	ChatGPT, used to generate treatment recommendations	Survey	ChatGPT showed 86.7% oncological management alignment with the multidisciplinary team.
Dergaa et al (2024) [49], Qatar	A simulation study	Mental health	ChatGPT, engaged as a digital psychiatric provider	Qualitative assessment	ChatGPT offered quick, empathetic, and guideline-concordant responses, whereas it struggled with clarification and customizing plans for complex scenarios.
Dergaa et al (2024) [50], Qatar	A simulation study	Hypertension, osteoarthritis, stress, diabetes, and asthma	ChatGPT 4.0, interacted with five hypothetical patient profiles to prescribe a 30-day fitness program	Qualitative assessment	While ChatGPT 4.0 can generate safety-conscious exercise programs, it lacks variability and cannot perform initial assessments or adjust regimens in real time.
Franco D’Souza et al (2023) [51], India	A simulation study	Psychiatric disorders	ChatGPT 3.5, interacted with 100 clinical case vignettes	Survey	ChatGPT 3.5 performed well in generating management strategies followed by diagnosis for psychiatric conditions.
Kianian et al (2024) [36], United States	A simulation study	Glaucoma	ChatGPT used to generate patient handouts	Survey	ChatGPT generated readable health information at a ninth-grade reading level and scored the quality of health resources with high precision (*r*=0.725; *P*<.001).
Lim et al (2024) [45], Singapore	A simulation study	Colorectal cancer	Retrieval-augmented generation-enhanced ChatGPT 4.0, instructed to provide colonoscopy screening recommendation	Survey	The enhanced model had higher accuracy in recommending colorectal screening intervals (79% vs 50.5%; *P*<.01) and experienced few hallucinations compared with the standard model.
Mondal et al (2023) [52], India	A simulation study	Lifestyle-related chronic diseases	ChatGPT 3.5, presented 20 cases of chronic disease management	Survey	ChatGPT 3.5 generated readable text with a mean FKRE^b^ score of 27.8 and had significantly higher accuracy (1.83, SD 0.37) and applicability (1.9, SD 0.21) than the hypothesized median score of 1.5.
Papastratis et al (2024) [53], Greece	A simulation study	Noncommunicable diseases	ChatGPT 3.5 and ChatGPT 4, interacted with 15 profiles to generate weekly meal plans.	Survey	ChatGPT 3.5 and 4.0 showed lower nutrient accuracy (81.5% and 81.6%) than a knowledge-based recommender (91%) but improved to 86% in ChatGPT 4.0 by inputting personalized energy target.
Pradhan et al (2024) [37], United States	A simulation study	Liver cirrhosis	ChatGPT 4.0, DocsGPT, Google Bard, and Bing Chat for generating a one-page patient education sheet	Survey	LLM-generated materials exhibited higher FKRE scores, 76%-99% accuracy rates, and comparable actionability to human-derived materials.
Puerto Nino et al (2024) [43], Canada	A simulation study	Benign prostate enlargement	ChatGPT 4.0+, fed with 88 queries for benign prostate enlargement	Survey	ChatGPT 4.0+ had a precision score range of 0.50-1 and a median general quality score of 4.
Seth et al (2023) [41], Australia	A simulation study	Carpal tunnel syndrome	ChatGPT (no version number), used to generate management strategies with six inquiries	Survey	ChatGPT accurately diagnosed carpal tunnel syndrome and recommended treatment options but faced challenges with erroneous references and insufficient information depth.
Singer et al (2024) [38], United States	A simulation study	Ophthalmology issues	Aeyeconsult powered by ChatGPT 4.0, interacted with 260 eyecare questions	Survey	Aeyeconsult outperformed ChatGPT 4.0 in accuracy (83.4% vs 69.2%) and demonstrated greater consistency in responses across repeated attempts on OphthoQuestions.
Spallek et al (2023) [42], Australia	A simulation study	Mental health and substance use disorders	ChatGPT 4.0 pro, interacted with queries for mental health and substance use	Survey	ChatGPT 4.0 had higher reading levels and accuracy but lacked human expert depth and breadth, with 23% featuring stigmatizing phrases.
Willms and Liu (2024) [44], Canada	An autoethnographic case study	Chronic disease prevention by increasing physical activity	ChatGPT 3.0, used to generate adaptive physical activity interventions	Qualitative assessment	ChatGPT 3.0 had acceptable accuracy and relevance in responding to prompts but sometimes provided false academic references.
Yang et al (2024) [39], United States	A case study	Diet management for preventing chronic illnesses	ChatDiet based on ChatGPT 3.5 Turbo to provide food recommendations	Causal graphs and qualitative assessment	ChatDiets effectively personalized food recommendations (85%-95% effectiveness) and demonstrated interactivity, but occasional hallucinations
Yeo et al (2023) [40], United States	A simulation study	Liver cirrhosis and hepatocellular carcinoma	ChatGPT Dec 15 version, entered with 164 questions for liver disease management	Survey with qualitative assessment	ChatGPT had 79.1% and 74% accuracy rates and provided emotional support; however, it might be unable to identify eligibility for hepatocellular carcinoma screening and liver transplantation.

^a^LLM: large language model.

^b^FKRE: Flesch-Kincaid reading ease score.

### Prompt Engineering and Wearable Devices Interacting With LLMs

Prompt engineering includes instructions, scenarios, queries, and output indicators. Instructions regarding the roles (eg, physician assistants) and tasks (eg, generating weekly meal plans) of LLMs were reported in two studies [45,53]. Real-world clinical cases [46] and imaginary patient scenarios [45,49,50,52,53] with medical profiles have been created to simulate health care management for chronic diseases, including diabetes, obesity, cardiovascular diseases, and mental health issues. These queries include real-time patient queries [21,47,48] and selected common queries from patients and their families [35,38,40,42,43]. These scenarios and queries interact with LLMs to generate responses related to the symptoms, complications, diagnoses, treatment, and management of chronic diseases. Output indicators, including word count, references, literacy levels, tone, format, and principles for generating management plans (eg, frequency, intensity, time, and type of exercises and diversity of meals) were applied to enhance the applicability of LLM recommendations [36,41,42,44,50].

Most importantly, Yang et al [39] explored the integration of ChatGPT with wearable devices to monitor physical activity levels, sleep patterns, and electrodermal activity, and update patient health profiles, allowing ChatGPT to adjust food recommendations dynamically. This integration allows the collection of patient data in real time and provides a dynamic and responsive health care management platform.

### Feasibility, Opportunities, and Challenges of LLMs Across the Chronic Disease Management Spectrum

#### Overview

LLMs are engaged in a spectrum of roles encompassing the prevention, screening, diagnosis, treatment, and long-term care of chronic diseases. Two studies have used LLMs to generate suggestions for increasing physical activity and nutrition-oriented food recommendations, thus contributing to the prevention of chronic diseases [39,44]. Lim et al [45] integrated LLMs to recommend screening and surveillance intervals for colorectal cancer and streamlined efforts for the early detection of chronic diseases. Three studies focused on treating chronic diseases by applying LLMs to generate treatment recommendations and support postoperative care [35,36,46]. In 14 studies, LLMs acted as digital health coaches, offered mental health support, managed symptoms, and generated diet and exercise plans to assist in long-term care of chronic diseases [21,37,38,40-43,47-53]. The feasibility, opportunities, and challenges inherent in these roles are described below.

#### Feasibility of LLMs in Managing Chronic Diseases

##### Relevance and Accuracy

The feasibility of LLMs in managing chronic diseases, including the relevance, accuracy, reliability, readability, and actionability of their responses, has been assessed by patients, caregivers, researchers, and health care specialists through interviews [21,47,48], content comparisons [46], grading [40,51], and measurements (eg, the Flesch-Kincaid Grade level) [36,37,52].

Averaging 92%, the LLMs demonstrated the ability to generate relevant recommendations, showing high pertinence to patient concerns [35,44]. LLMs also have acceptable accuracy in identifying diagnoses and deterioration of symptoms, recommending investigations and treatment options, and generating health educational materials for several chronic diseases (including carpal tunnel syndrome, liver cirrhosis, and mental health) [41,42], with rates ranging from 76% to 99% as validated by health care experts [37]. Additionally, LLMs demonstrated high concordance rates with various guidelines, including those for the postoperative care of hearing loss (100%) [35], multidisciplinary tumor board recommendations (86.7%) [46], and the creation of general exercise programs that adhere to the rate of perceived exertion guidelines and research evidence [50]. Two studies further corroborated these findings, indicating that ChatGPT exceeded the hypothesized median scores for accuracy in addressing queries related to chronic diseases (eg, obesity and diabetes) [52], albeit with occasional issues regarding the accuracy of the cited references [44].

In contrast, Yeo et al [40] revealed the mixed performance of LLMs, identifying 50% mixed or incorrect responses in screening, diagnosing, and managing hepatocellular carcinoma. In particular, LLMs failed to correctly identify eligibility and screening tests for hepatocellular carcinoma based on patient characteristics (eg, age) and failed to determine cut-offs for specific conditions, such as liver transplantation [40]. Similarly, in colorectal cancer screening, ChatGPT 4.0 experienced hallucinations in identifying high-risk features of colorectal cancer and recommended incorrect screening intervals in 51% of cases [45]. LLMs may be inaccurate when performing complex cancer screening tasks.

Further comparative analyses revealed that the retrieval-augmented generation-enhanced LLMs outperformed the general-purpose LLMs in terms of response accuracy. For example, retrieval-augmented generation-enhanced ChatGPT 4.0 resulted in few hallucinations and significantly outperformed its predecessor in correctly recommending colorectal cancer screening intervals (79% vs 50.5%) [45] and responding to ophthalmological questions (83.4% vs 69.2%) [38].

**Figure 3:**
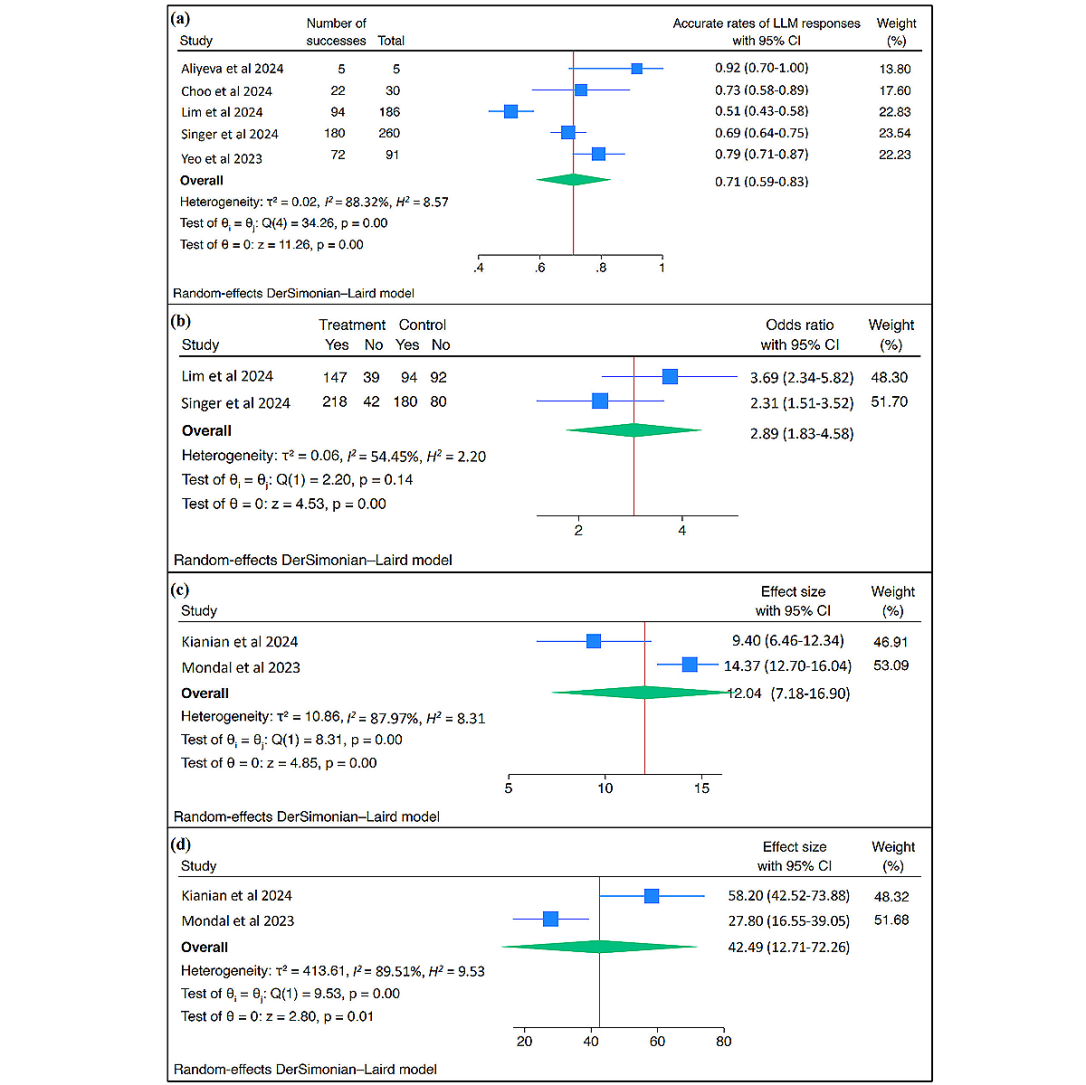
Forest plot: (a) pooled accurate rate of LLMs, (b) pooled accurate rate of retrieval-augmented generation-enhanced LLMs compared to general-purpose LLMs, (c) pooled effect sizes of the Flesch-Kincaid Grade Level score, and (d) the Flesch-Kincaid Reading Ease Score. LLM: large language model.

The random-effects meta-analysis of the aforementioned studies [35,38,40,45,46] showed a pooled accuracy rate of 71% (95% CI 0.59-0.83; *I^2^*=88.32%; *P*<.001; Figure 3A). Sensitivity analysis using the leave-one-out approach revealed that the removal of individual studies from the pooled accuracy rate was not statistically significant (Figure S1 in Multimedia Appendix 4 [21,36-53]). Compared to general-purpose LLMs, retrieval-augmented generation-enhanced LLMs had a higher rate of accurate responses to colorectal cancer screening and ophthalmology questions (odds ratio 2.89, 95% CI 1.83-4.58; *I^2^*=54.45%; *P*<.001; Figure 3B) [38,45].

##### Reliability

The reliability of LLM performance remains a major concern. Although retrieval-augmented generation-enhanced LLMs, such as Aeyeconsult, demonstrated improved response reliability compared to general-purpose LLMs (eg, ChatGPT 4.0), these models still experienced missing, multiple, or contradictory answers [38]. These inconsistencies often stem from failures within the underlying LLM architecture, information retrieval processes, or the synthesis of information from multiple sources [38]. Studies have also noted instances of hallucinations, citations of nonexistent sources, and insufficient depth of information [38,41]. Qualitative feedback from patients further emphasizes the unreliability of LLM-generated information, citing concerns about outdated data, biases in training datasets, and LLMs’ self-acknowledged limitations in verifying factual accuracy [21,48].

##### Readability

The readability of LLM responses was consistently rated high across the included studies (67% to 98%) [35-37,42,52]. LLMs outperform web pages [36] and human-derived materials [37] in terms of comprehension, in which health care recommendations regarding cirrhosis, obesity, diabetes, and cardiovascular diseases are easily understood by individuals with high school or higher educational levels [37,52]. The random-effects meta-analysis of the two included studies [36,52] indicated that the Flesch-Kincaid Grade Level score and Flesch-Kincaid Reading Ease Score were 12.04 (95% CI 7.18-16.90; *I^2^*=87.97%; *P*<.001; Figure 3C) and 42.49 (95% CI 12.71-72.26; *I^2^*=89.51%; *P*=.01; Figure 3D), respectively.

##### Actionability

The actionability of the LLM responses remains unclear. While Pradhan et al [37] reported no significant differences between LLM-derived and human-derived cirrhosis management materials concerning actionability, only the human-derived content met the actionable score threshold of ≥70%. The LLM responses may lack depth and details for practical application [41].

#### Opportunities of LLMs in Managing Chronic Diseases

##### Increasing Knowledge and Awareness

Using internet-enabled devices, LLMs provide equal and free access to chronic disease information, especially for patients from rural areas [21,47]. This utility enhances patient knowledge and awareness of ailments, preventive measures, symptoms, and the management of chronic diseases, including cancer, diabetes, and kidney failure [21,47,48]. This also helped dispel misperceptions about lifestyle modifications (eg, diet and smoking cessation) and chemotherapy in cancer management [21]. As noted by the participants, this benefit is particularly pronounced compared with traditional search engines, which require navigating multiple websites for consolidated information [47].

##### Promoting Self-Management Behaviors

By motivating health goals and developing achievable plans, LLMs promote patient self-management behaviors, including diets, smoking cessation, physical activities, sleep, and meditation [47,48]. LLMs also inform patients about using nonpharmacological techniques, such as relaxation, sleep hygiene practices, and stress-reduction techniques, to cope with symptoms of chronic diseases, including insomnia, fatigue, nausea, and pain [21,48]. However, a major limitation is the current inability of LLMs to store and manage long-term behavioral change data. The integration of LLMs with eHealth systems, wearables, and health management applications for continuous monitoring and tracking of health conditions (eg, blood glucose level) aids in facilitating personalized care plans and setting reminders for health behaviors (eg, taking medication) [47].

##### Enhancing Emotional, Social, and Health Care Support

LLMs provide a nonjudgmental space for emotional expression and offer compassionate responses to enhance patients’ emotional well-being [48]. Yeo et al [40] highlighted ChatGPT’s psychological and practical support for patients following the diagnosis of hepatocellular carcinoma. LLMs also help patients practice cognitive behavioral therapy techniques, which involve identifying negative thoughts and replacing them with more balanced thoughts to positively reframe their emotions [48]. However, some critical concerns persist, such as a lack of capability in assessing mental health conditions [48] and patients’ perceived lack of deep understanding and personalized empathy compared with human health care professionals [47].

At the social level, LLMs demonstrated the capability to guide patients in accessing hotlines, counselors, and web-based support groups, such as cancer support groups, which are important for connecting with other patients, attaining peer support, and accessing updated treatment information regarding chronic diseases [21,47,48].

At the health care level, LLMs improve health care support by linking health resources [48] and providing scalable support accessible to a large number of patients simultaneously [47]. Many patients reported difficulties in securing appointments with specialists for their chronic diseases and were dissatisfied with the limited time they had with specialists to understand their conditions, preventive measures, and treatment procedures [47]. LLMs alleviated this issue by offering comprehensive information on various chronic conditions, thus decreasing the need for frequent specialist consultations, helping patients become more self-reliant and better informed about their health, thereby reducing the strain on the health care system and improving overall patient outcomes [47,48].

#### Challenges of LLMs in Managing Chronic Diseases

##### Potential Privacy, Language, and Cultural Issues

Privacy and security concerns are paramount for patients when using LLMs for chronic disease management [21,47,48]. Patients reported the absence of data protection guidelines and a lack of anonymous features on LLMs because most tools require registration. Patients were unconfident about sharing their personal health data and feared potential misuse. Additionally, although LLMs can manage basic linguistic tasks, they often fail to grasp dialectal subtleties [47,48]. Spallek et al [42] reported that 23% of LLM outputs had at least one stigmatizing phrase. This inadequacy is evident in the context of traditional medicine, where culturally rooted concepts are not effectively understood, potentially leading to misinformation [47]. This issue underscores the need for further cultural sensitivity training for LLMs to ensure that they can handle diverse linguistic and cultural contexts accurately and respectfully.

##### Incompetence in Tackling Advanced Tasks in Chronic Disease Management

LLMs are not sufficiently mature to address advanced chronic disease management tasks. Some LLMs cannot interpret complex diagnostic reports that include nontext inputs such as radiology images, blood tests, and other medical documents [47,48]. LLMs rely on patients’ self-reported symptoms to diagnose diseases, and they cannot initiate dialogues, which precludes them from probing hidden symptoms, clarifying patient conditions, and identifying appropriate management plans [42,47-49]. LLMs are also unreliable because they provide simplified recommendations, frequently acknowledge potential inaccuracies in addressing complex comorbidities of chronic diseases, and recommend effective medicines based on patient data [21,47,48].

##### Gaps in Generating Personalized Chronic Disease Management Regimens

Regarding content and format, LLMs cannot generate personalized chronic disease management regimens. For example, LLMs were unable to monitor individuals’ physiological responses, failed to adjust physical exercise regimens in real time, and could not generate customized treatment plans in complex disease scenarios (eg, systematic lupus erythematosus) [21,50]. In contrast, Yang et al [39] integrated ChatGPT with wearable devices to monitor patient physical activity, sleep patterns, and electrodermal activity to update health profiles in real time, allowing ChatGPT to dynamically adjust food recommendations. The integration of LLMs with wearables can address this challenge by providing a dynamic and responsive platform for chronic disease management. Moreover, LLMs may struggle to transform text-based information into multimodal formats (eg, images and videos), influencing the effective delivery of information tailored to patient preferences [21].

### Quality of Studies

Tables S1 and S2 in Multimedia Appendix 4 [21,36-53] present the results of the quality assessment. Three quasi-experimental studies had a serious risk of bias due to potential covariates and deviations from intended interventions (eg, access to health care information from other web-based resources) [21,47,48]. A total of 17 simulation and case studies attained methodology quality scores ranging from 66.7% to 89.6%, which were influenced primarily by the lack of valid instruments for measuring the feasibility of LLMs [35,51,53]; inadequate reporting of qualitative data collection, coding, and analysis processes [44,49,50]; and having small samples of patient scenarios mimicking chronic disease health care seeking, ranging from 5 to 30 in ten studies [35-37,41,42,46,49,50,52,53].

## Discussion

### Principal Findings

This systematic review included 20 studies to synthesize evidence on the feasibility, opportunities, and challenges of LLMs in the management of chronic diseases. Findings suggested that LLMs can feasibly recommend relevant, comprehensible, and accurate health information (71%, 95% CI 0.59-0.83; *I^2^*=88.32%; *P*<.001). They enhanced equitable information access, patient awareness, and self-management behaviors and provided emotional support, social connections, and health care resource linkages, collectively contributing to improving chronic disease outcomes. Nevertheless, LLMs face challenges in addressing privacy, language, and cultural issues; undertaking advanced diagnostic and medication recommendation tasks; and generating personalized regimens with real-time adjustments and multiple modalities. These insights are pivotal for health care professionals to harness the transformative potential of LLMs for chronic disease management.

Feasibility, which encompasses relevance, accuracy, and reliability, is the premise for the application of LLM in chronic disease management. Consistent with previous studies [14], LLMs exhibit the capacity to generate relevant responses tailored to the concerns of patients with chronic diseases [35,44]. This adaptability is attributed to their advanced natural language processing abilities, which enable them to align closely with patient inquiries and medical contexts [15,16]. However, LLMs presented a mixed profile of accuracy across different tasks of chronic disease management, with a pooled accuracy rate of 71%. Specifically, LLMs have shown acceptable accuracy (76%-99%) in generating health educational materials; however, their accuracy is particularly concerning when applied to cancer screening tasks [40,45]. To enhance accuracy, several studies enhanced LLMs using retrieval-augmented generation to combine LLMs with contextual knowledge bases [38,39,45]. Compared with general-purpose models, retrieval-augmented generation-enhanced LLMs exhibit greater accuracy and adhere more closely to medical guidelines in response to colorectal cancer screening and ophthalmology questions [45]. This can be attributed to the integration of domain-specific knowledge through specialized datasets and clinical guidelines [38]. This approach provides LLMs with a deeper contextual understanding of medical terminology and ensures a clinically sound response [39]. In addition, iterative feedback from clinical experts further refines these models, enhancing their precision in tasks such as disease risk assessment, diagnostic accuracy, and guideline-based recommendations [17]. Despite these advancements, issues such as hallucinations, contradictory responses, and citations of nonexistent sources persist [41]. Furthermore, the tendency of LLMs to provide simplified recommendations, acknowledge potential inaccuracies, and advise users to consult health care professionals diminishes their reliability [21,47,48]. Therefore, integrating LLMs into chronic disease management requires ongoing development, clinical validation, and collaboration with health care professionals to optimize their accuracy and reliability.

This review confirms that LLMs provide multifaceted opportunities for chronic disease management at individual, social, and health care system levels. At the individual level, LLMs provide equitable access to health information about chronic diseases, particularly benefiting patients residing in rural areas who may have limited access to health information and lower health literacy [21,47]. Consistent with this review, previous literature has also highlighted that LLMs facilitate health communication through telehealth, minimizing geographical, travel-related, and financial challenges, and providing a solution to disparities in health care information access among rural communities [24]. In rural and remote settings, policy makers should strategically integrate LLMs into health care systems to ensure equitable access to health information, resources, and web-based support. This approach is crucial for reducing the disparities in chronic disease management among underserved populations. More importantly, such accessibility can enhance patient knowledge and awareness of preventive measures, diagnoses, symptoms, treatment, and management strategies for various chronic diseases, including cancer, diabetes, and kidney failure [21,47,48]. In line with the findings of this review, previous reviews have confirmed that LLMs play a significant role in patient health education by generating health education materials and providing multifaceted recommendations covering medical information, lifestyle recommendations, and perioperative care instructions [54,55]. Advanced algorithms and extensive dataset training of LLMs may enable natural language interactions, real-time feedback, and personalized health information, effectively addressing patient queries and improving patients’ knowledge of chronic diseases [18]. These characteristics make LLMs more advantageous for health education than traditional algorithm-based applications, which typically rely on tedious checkbox questionnaires, offer constrained responses, and require backend processing from health care professionals [56,57].

Additionally, this review reveals the burgeoning interest in leveraging LLMs to enhance behavior-changing interventions. The included studies demonstrated that LLMs positively influenced patient adherence to recommended healthy behaviors, including a balanced diet, regular exercise, and smoking cessation [47]. LLMs also show promise in assisting patients with disease monitoring and self-management of physical (eg, fatigue and pain) and emotional (eg, fear and anxiety) symptoms by recommending practical tips and psychotherapeutic exercises, such as guided imagery [21,48]. The findings are consistent with previous research indicating the effectiveness of LLMs in promoting health-related behavioral changes by enhancing health knowledge, debunking health myths, and providing motivational support [54,58]. However, a critical limitation of the current literature is the insufficient integration of established behavior-change theories within LLM-based interventions. This methodological gap limits the efficiency of intervention and hinders the identification of causal mechanisms. To optimize LLM efficacy in facilitating behavioral change, theoretical models should be integrated to tailor content precisely and maximize the likelihood of sustained behavioral changes [44].

Although LLMs show promise in enhancing emotional support for patients with chronic diseases, their capacity for genuine empathy remains controversial. Several of the included studies reported positive patient experiences with LLM-provided emotional support [21,40]; however, it might lack empathy and personalized support compared with human health care professional interactions [47,49]. In line with the previous literature [27], existing algorithms for LLMs, while capable of emulating empathetic responses and offering practical advice, may not fully grasp the complexities of human emotions. Therefore, an integrative strategy that leverages the strengths of LLMs while preserving the irreplaceable human touch should be implemented in future practice for chronic disease management.

At the social level, LLMs can guide patients to support their networks and facilitate peer and social support. Previous studies corroborate this finding, indicating that LLMs can support patients by connecting them with relevant resources [18,59]. At the health care level, the findings suggest that LLMs can deliver scalable health support [47], link medical resources, and recommend crisis interventions [48] to improve health care support and reduce patients’ reliance on face-to-face consultations at health care facilities. These findings align with previous literature, suggesting that broader implementation of LLMs could alleviate the burden on health care systems [28,60]. Nonetheless, the positive effects of LLMs have only been tested among a small cohort of patients [21,47,48], and rigorous randomized controlled trials are needed to validate these outcomes.

However, there are several challenges to the application of LLMs for managing chronic diseases. First, uncertainty remains regarding data privacy and sharing of conflicts when using LLMs for chronic disease management. LLMs use personal data to provide accurate and customized recommendations. However, this requirement often conflicts with stringent privacy protocols. Owing to a lack of robust data protection guidelines and the inability to register anonymously, patients may fear data leakage or misuse and hesitate to share personal health information [47,48]. Studies have integrated wearable devices with LLMs to collect real-time data, such as sleep patterns [39], which complicates issues related to data encryption and security during transmission [60]. Hence, robust data-protection measures should be implemented to safeguard patient information. Advanced anonymization techniques and end-to-end encryption protocols should be used to protect patient privacy and secure data transmission while interacting with LLMs.

Second, the ethical implications of using LLMs to diagnose chronic diseases must be clarified. Although studies have shown that LLMs can accurately diagnose conditions such as psychiatric disorders [51] and carpal tunnel syndrome [41], their comprehensive diagnostic capabilities remain limited. As noted in the included studies [42,47-49], some LLMs rely on patients’ self-reported symptoms and general medical knowledge, and cannot perform physical examinations or exactly interpret complex diagnostic reports, such as radiology images and blood tests. This may lead to inaccurate or incomplete diagnoses [40] and delay necessary treatments. The presence of biases in training datasets can also lead to skewed recommendations, potentially jeopardizing patient safety [21,48]. Therefore, ethical standards should be considered carefully before integrating LLMs into health care systems to perform diagnostic tasks. It has been suggested that LLMs be fine-tuned with domain-specific knowledge to ensure that they align with the most current medical standards and practices for chronic diseases. Ideally, multimodal LLMs capable of integrating diverse data modalities, such as radiology images and laboratory data, should be developed to improve diagnostic precision. Addressing this ethical concern is crucial to upholding the trustworthiness of LLMs for chronic disease management.

Another challenge is that LLM recommendations cannot achieve adequate personalization of information content and modality. Despite the potential of LLMs in personalized health care based on their natural language processing capabilities [24,27], significant gaps remain in achieving genuine personalization. For complex conditions such as cancer [21] and systemic lupus [50], LLMs tend to provide generic and broad advice and struggle to make real-time adjustments to personalized regimens (eg, exercises) [49]. The integration of wearables with LLMs may increase the provision of dynamic and personalized health recommendations. Wearable devices can continuously monitor vital physiological parameters such as heart rate, blood pressure, glucose levels, and physical activity. These real-time data can be fed into LLMs to adjust treatment plans timeously and offer highly personalized health care interventions. However, achieving this integration involves technical hurdles, including ensuring instantaneous data processing, implementing advanced data fusion techniques to integrate diverse data streams, and maintaining robust data accuracy. In addition, stringent data security measures must be implemented to ensure patient confidentiality. Moreover, some LLMs (eg, ChatGPT 3.0) operate primarily in text-based formats and do not readily generate multimodal information to effectively deliver information tailored to patient preferences [21]. Their inability to process and generate multimodal information, such as images, videos, and other nontextual medical data, limits their efficacy in personalized chronic disease management. Additional interdisciplinary collaborations between clinical experts and artificial intelligence professionals coupled with rigorous clinical trials are imperative to ensure the effective integration of LLMs into chronic disease management frameworks.

LLMs optimize chronic disease management by enhancing patient awareness and self-management behaviors, as well as providing emotional support, social connections, and health care resource linkages. However, key challenges, including privacy issues, inconsistent accuracy, and a lack of personalization in LLM recommendations, should be addressed. A multifaceted approach incorporating robust data security, domain-specific model fine-tuning, multimodal data integration, and wearables is essential to address these challenges and realize the potential of LLMs in chronic disease management.

### Limitations

This study had several limitations. First, although this review searched 11 databases, our search strategy did not include keywords representing various chronic diseases and may have missed relevant studies. Additionally, most of the searched studies were conducted in high-income countries (12/20, 60%), which may limit the generalizability of the findings. Although two Chinese databases were searched, no relevant studies were identified, highlighting the need for increased efforts to leverage LLMs for chronic disease management in China and other developing regions. Second, heterogeneity was observed in the pooled analyses of LLM feasibility outcomes, including accuracy rates and readability. Heterogeneity could potentially undermine confidence in the synthesized findings, necessitating a conservative interpretation of the results. Third, most of the studies (n=17) included in this review relied on simulations rather than real-world implementations. The use of simulated data raises concerns regarding the generalizability of the results to actual clinical settings, particularly given the small sample size of patient scenarios. Additionally, biases, such as invalid outcome measurements, may exist within the included studies, which could further affect the validity of the findings. To address these limitations, future research should explore patients’ experiences with LLMs in chronic disease management through rigorous randomized controlled trials to ensure a more robust and representative assessment of their real-world applicability.

### Conclusions

This review encompasses 20 publications and synthesizes the evidence of the transformative potential of LLMs in chronic disease management. LLMs have demonstrated the feasibility of generating relevant, comprehensible, and accurate health information, although their reliability and actionability remain controversial. They presented opportunities to (1) enhance patient knowledge and awareness of chronic diseases; (2) facilitate self-management behaviors in lifestyle modification and symptom coping; and (3) enhance emotional, social, and health care support. However, LLMs face challenges in addressing privacy and cultural issues, performing advanced diagnostic and medication tasks, and generating personalized regimens. Further empirical validation is crucial for transforming LLMs into invaluable adjuncts for health care professionals to improve chronic disease management.

## Data Availability

All data generated or analyzed during this review are included in this published article and the Multimedia Appendices; additional data are available from the corresponding author upon reasonable request.

## Appendix

Multimedia Appendix 1 PRISMA 2020 checklist.

Multimedia Appendix 2 Search keywords and search strategy in each database.

Multimedia Appendix 3 A detailed overview of the study characteristics.

Multimedia Appendix 4 Results of sensitivity analysis and study quality assessment.

## Abbreviations

LLM: large language model
PICOS: Population-Intervention-Comparator-Outcomes-Study
PRISMA: Preferred Reporting Items for Systematic Reviews and Meta-Analysis
