# Supplementary material for: Unveiling the Potential of Large Language Models in Transforming Chronic Disease Management: Mixed Methods Systematic Review
Source: J Med Internet Res. 2025 Apr 16;27:e70535. doi: 10.2196/70535 (PMC12044321; doi:10.2196/70535)
Supplement: Multimedia Appendix 2 [file jmir_v27i1e70535_app2.docx]

**Table S1** Key search teams

| **Key concepts** | **Key search terms** |
| --- | --- |
| #1 Large Language Model | "Large language model*" or "multimodal AI" or "large multimodal model*" or "natural language process*" or "natural language generat*" or "machine learning" or ChatGPT or GPT or "Google Bard" or OpenAI or "generative pre-trained transformer*" or "generative AI" or chatbot* or chatterbot or chatter robot or AI agent or GenAI or "artificial intelligence" |
| #2 Chronic disease management outcomes | "Health behavior*" or "healthy behaviour*" or "behavior change" or "behaviour change" or "healthcare seek* behavior*" or "healthcare seek* behaviour*" or "health promot*" or health knowledge or health attitude* or beliefs or "health educat*" or "health practice*" or patient adheren* or self-manag* or "health manag*" or "lifestyle modification" or treatment adhere* or patient compliance or self care |

**Table S2** Search Strategy in Each Database

| **Cochrane Central Register of Controlled Trials**  **April 17, 2024** | | |
| --- | --- | --- |
| #1 | MeSH descriptor: [Natural Language Processing] explode all trees | 69 |
| #2 | MeSH descriptor: [Artificial Intelligence] explode all trees | 3,166 |
| #3 | MeSH descriptor: [Machine Learning] explode all trees | 975 |
| #4 | (Large language model* or multimodal AI or large multimodal model* or natural language process* or natural language generat* or machine learning or ChatGPT or GPT or Google Bard or OpenAI or generative pre-trained transformer* or generative AI or chatbot* or chatterbot or chatter robot or AI agent or GenAI or artificial intelligence):ti,ab,kw | 8,697 |
| #5 | MeSH descriptor: [Health Behavior] explode all trees | 47,723 |
| #6 | MeSH descriptor: [Health Promotion] explode all trees | 8,910 |
| #7 | MeSH descriptor: [Patient Compliance] explode all trees | 15,445 |
| #8 | MeSH descriptor: [Self-Management] explode all trees | 1,237 |
| #9 | MeSH descriptor: [Self Care] explode all trees | 7,800 |
| #10 | MeSH descriptor: [Patient Education as Topic] explode all trees | 10,833 |
| #11 | MeSH descriptor: [Treatment Adherence and Compliance] explode all trees | 37,439 |
| #12 | (((Health* and (behavior* or behaviour* or seek* or seek* behavior or seek* behaviour or promot* or knowledge or attitude* or belief* or educat* or manag*)) or patient adherence or self-manag* or lifestyle modification or treatment adhere* or patient complian* or self care)):ti,ab,kw (Word variations have been searched) | 276,950 |
| #13 | #1 OR #2 OR #3 OR #4 | 10,481 |
| #14 | #5 OR #6 OR #7 OR #8 OR #9 OR #10 OR #11 OR #12 | 297,224 |
| #15 | #13 AND #14 | 2,194 |
|  | Limit to Trials | 1,891 |
| **CINAHL**  **April 17, 2024** | | |
| #1 | TI (“large language model*" or "multimodal AI" or "large multimodal model*" or "natural language process*" or "natural language generat*" or "machine learning" or ChatGPT or GPT or "Google Bard" or OpenAI or "generative pre- trained transformer*" or "generative AI" or chatbot* or chatterbot or chatter robot or AI agent or GenAI or "artificial intelligence") | 10,926 |
|  |  |  |
| #2 | MH health behavior | 40,943 |
| #3 | MH health promotion | 50,468 |
| #4 | MH patient compliance | 21,949 |
| #5 | MH self- management | 2,205 |
| #6 | MH self care | 25,559 |
| #7 | MH patient education | 37,986 |
| #8 | TI (((Health* and (behavior* or behaviour* or seek* or seek* behavior or seek* behaviour or promot* or knowledge or attitude* or belief* or educat* or manag*)) or "patient adherence" or self-manag* or "lifestyle modification" or "treatment adhere*" or "patient complian*" or self care)) | 63,410 |
| #9 | MH natural language processing | 2,342 |
| #10 | MH artificial intelligence | 7,627 |
| #11 | #1 OR #9 OR #10 | 17,160 |
| #12 | #2 OR #3 OR #4 OR #5 OR #6 OR #7 OR #8 | 197,443 |
| #13 | #11 AND #12 | 338 |
| **Embase**  **April 17, 2024** | | |
| #1 | ‘large language model’:abti OR ‘multimodal ai’:ab, ti OR ‘large multimodal modal*’:ab,ti OR ‘natural language process*’:ab,ti OR ‘natural language generat*’:ab,ti OR ‘machine learning’:ab,ti OR chatgpt:ab,ti OR gpt:ab,ti OR ‘google bard’:ab,ti OR openai:ab,ti OR ‘generative pre-trained transformer’:ab,ti OR ‘generative ai’:ab,ti OR chatbot*:ab,ti OR chatterbot:ab,ti OR ‘chatter robot’:ab,ti OR ‘ai agent’:ab,ti OR genai:ab,ti OR ‘artificial intelligence’:ab,ti | 169,746 |
| #2 | health*:ti AND (behavior*:ti OR behaviour*:ti OR seek*:ti OR ‘seek* behavior’:ti OR ‘seek’ behaviour:ti OR promot*:ti OR knowledge:ti OR attitude*:ti OR belief*:ti OR educat*:ti OR manag*:ti) OR ‘patient adherence’:ti OR ‘self manag*’:ti OR ‘lifestyle modification’:ti OR ‘treatment adhere’:ti OR ‘patient complian*’:ti OR ‘self care’:ti | 160,218 |
| #3 | #1 AND #2 | 607 |
| **IEEE Xplore**  **April 17, 2024** | | |
| #1 | ("Full Text & Metadata":Large language model* or natural language process* or natural language generat* or ChatGPT ) AND ("Full Text & Metadata":health) | 496 |
| **Medline via Ovid**  **April 17, 2024** | | |
| #1 | exp Natural Language Processing/ | 6,574 |
| #2 | exp Artificial Intelligence/ | 193,811 |
| #3 | exp Nerual Networks, Computer/ | 66,009 |
| #4 | (large language model* or multimodal AI or large multimodal model* or natural language process* or natural language generat* or machine learning or ChatGPT or GPT or Google Bard or OpenAI or generative pre-trained transformer* or generative AI or chatbot* or chatterbot or chatter robot or AI agent or GenAI or artificial intelligence).ab,ti. | 143,328 |
| #5 | exp Health Behavior/ | 366,183 |
| #6 | exp Health Knowledge, Attitudes, Practice/ | 128,207 |
| #7 | exp Health Promotion/ | 86,558 |
| #8 | exp Patient Compliance/ | 86,530 |
| #9 | exp Self-management/ | 5,814 |
| #10 | exp Self-Care/ | 63,436 |
| #11 | exp Patient Education as Topic/ | 88,815 |
| #12 | exp “Treatment Adherence and Compliance”/ | 279,147 |
| #13 | ((Health* and (behavior* or behavior* or seek* or seek* behavior or seek* behaviour or promot* or knowledge or attitude* or belief* or educat* or manag*)) or patient adherence or self-manag* or lifestyle modification or treatment adhere* or patient complian* or self care).ab,ti. | 1,319,033 |
| #14 | 1 or 2 or 3 or 4 | 273,396 |
| #15 | 5 or 6 or 7 or 8 or 9 or 10 or 11 or 12 or 13 | 1,749,319 |
| #16 | #14 AND #15 | 15,174 |
| #17 | limit 16 to humans | 9,981 |
| #18 | limit 17 to full text | 2,272 |
| **ProQuest Health & Medicine Collection**  **April 17, 2024** | | |
| #1 | mainsubject(natural language processing) | 3,693 |
| #2 | mainsubject.Exact("large language models") | 152 |
| #3 | mainsubject.Exact("natural language processing") | 3,566 |
| #4 | mainsubject.Exact("agents (artificial intelligence)" OR "artificial intelligence") | 41,027 |
| #5 | mainsubject.Exact("artificial neural networks" OR "neural networks (computer)" OR "neural networks") | 63616 |
| #6 | title(Large language model* or multimodal AI or large multimodal model* or natural language process* or natural language generat* or machine learning or ChatGPT or GPT or Google Bard or OpenAI or generative pre-trained transformer* or generative AI or chatbot* or chatterbot or chatter robot or AI agent or GenAI or artificial intelligence) OR abstract(Large language model* or multimodal AI or large multimodal model* or natural language process* or natural language generat* or machine learning or ChatGPT or GPT or Google Bard or OpenAI or generative pre- trained transformer* or generative AI or chatbot* or chatterbot or chatter robot or AI agent or GenAI or artificial intelligence) | 65,941 |
| #7 | mainsubject.Exact("health behavior") | 37,225 |
| #8 | mainsubject.Exact("health knowledge, attitudes, practice") | 13,512 |
| #9 | mainsubject.Exact("health promotion") | 38,230 |
| #10 | mainsubject.Exact("patient compliance") | 16,757 |
| #11 | mainsubject.Exact("self care") | 4,554 |
| #12 | mainsubject.Exact("patient education") | 12,940 |
| #13 | (abstract(((Health* and (behavior* or behaviour* or seek* or seek* behavior or seek* behaviour or promot* or knowledge or attitude* or belief* or educat* or manag*)) or "patient adherence" or self- manag* or "lifestyle modification" or "treatment adhere*" or "patient complian*" or self care)) OR title(((Health* and (behavior* or behaviour* or seek* or seek* behavior or seek* behaviour or promot* or knowledge or attitude* or belief* or educat* or manag*)) or "patient adherence" or self- manag* or "lifestyle modification" or "treatment adhere*" or "patient complian*" or self care))) AND mainsubject.Exact("patient education") | 4,791 |
| #14 | #1 OR #2 OR #3 OR #4 OR #5 OR #6 | 132,674 |
| #15 | #7 OR #8 OR #9 OR #10 OR #11 OR #12 OR #13 | 111,994 |
| #16 | #14 AND #15 | 503 |
| **ScienceDirect**  **April 17, 2024** | | |
| #1 | (large language model) AND health | 445 |
| **Scopus**  **April 17, 2024** | | |
| #1 | Article title, Abstract, Keywords ( "Large language model*" OR "multimodal AI" OR "large multimodal model*" OR "natural language process*" OR "natural language generat*" OR "machine learning" OR chatgpt OR gpt OR "Google Bard" OR openai OR "generative pre-trained transformer*" OR "generative AI" OR chatbot* OR chatterbot OR chatter AND robot OR ai AND agent OR genai OR "artificial intelligence" ) | 47,265 |
| #2 | Article title, Abstract, Keywords ( ( health* AND ( behavior* OR behaviour* OR seek* OR seek* AND behavior OR seek* AND behaviour OR promot* OR knowledge OR attitude* OR belief* OR educat* OR manag* ) ) OR "patient adherence" OR self-manag* OR "lifestyle modification" OR "treatment adhere*" OR "patient complian*" OR "self care" ) | 1,203,661 |
| #3 | #1 AND #2 | 907 |
|  | Exclude (Review or Conference paper or Book chapter OR Conference review or Letter) | 425 |
| **Web of Science Core Collection**  **April 17, 2024** | | |
| #1 | "Large language model*" or "multimodal Al" or "large multimodal model*" or  "natural language process*" or "natural language generat*" or "machine learning" or ChatGPT or GPT or "Google Bard" or OpenAl or "generative pre-trained transformer*" or "generative Al' or chatbot* or chatterbot or chatter robot or Al agent or GenAl or "artificial intelligence" (Topic) | 551,306 |
| #2 | (Health* and (behavior* or behaviour* or seek* or seek* behavior or seek* behaviour or promot* or knowledge or attitude* or belief* or educat* or manag*)) or "patient adherence" or self-manag* or "lifestyle modification" or "treatment adhere*" or "patient complian*" or self care) (Title) | 165,984 |
| #3 | #1 AND #2 | 1,208 |
| #4 | Meeting Abstract or Editorial Material or Retraction or Letter or Retracted Publication or Book Chapters or Book or Correction or Data Paper or News Item (Exclude-Document Types) | 1,096 |
| **China National Knowledge Internet**  **April 17, 2024** | | |
| #1 | (主题：大模型 + 大语言模型 + 多模态 + 跨模态 + 自然语言处理 + 自然语言生成 + ChatGPT + GPT + Google Bard + OpenAI +生成式预训练 Transformer + 生成式AI + 聊天机器人) AND (主题：健康行为 + 健康促进 + 健康 + 健康知识 + 健康教育 + 健康信念 + 依从性 + 自我管理) limit to 学术期刊 | 242 |
| **Sinomed**  **April 17, 2024** | | |
| #2 | "大模型"[核心字段:智能] OR "大语言模型"[核心字段:智能] OR "多模态"[核心字段:智能] OR "跨模态"[核心字段:智能] OR "自然语言处理"[核心字段:智能] OR "自然语言生成"[核心字段:智能] OR "ChatGPT"[核心字段:智能] OR "生成式预训练"[核心字段:智能] AND "Transformer"[核心字段:智能] OR "聊天机器人"[核心字段:智能] | 3064 |
| #3 | "健康行为"[不加权:扩展] | 49624 |
| #4 | "健康促进"[不加权:扩展] | 6550 |
| #5 | "自然语言处理"[不加权:扩展] | 440 |
| #6 | "健康知识, 态度, 实践"[不加权:扩展] | 10261 |
| #7 | "治疗依从性"[不加权:扩展] OR "服药依从性"[不加权:扩展] | 40754 |
| #8 | "自我管理"[不加权:扩展] | 10377 |
| #9 | "健康教育"[不加权:扩展] OR "健康教育"[不加权:扩展] | 113157 |
| #10 | "健康行为"[核心字段:智能] OR "健康促进"[核心字段:智能] OR "健康"[核心字段:智能] OR "健康知识"[核心字段:智能] OR "健康教育"[核心字段:智能] OR "健康信念"[核心字段:智能] OR "依从性"[核心字段:智能] OR "自我管理"[核心字段:智能] | 330547 |
| #11 | (#10) OR (#9) OR (#8) OR (#7) OR (#6) OR (#4) OR (#3) | 330547 |
| #12 | (#5) OR (#2) | 3064 |
| #13 | #11 AND #12 | 76 |
