# Supplementary material for: Unveiling the Potential of Large Language Models in Transforming Chronic Disease Management: Mixed Methods Systematic Review
Source: J Med Internet Res. 2025 Apr 16;27:e70535. doi: 10.2196/70535 (PMC12044321; doi:10.2196/70535)
Supplement: Multimedia Appendix 3 [file jmir_v27i1e70535_app3.docx]

**Table S1** A detailed overview of the study characteristics

| **Author, year, and country** | **Study design** | **Chronic health conditions** | **Characteristics of LLMs^a^** | **Outcome assessment** | **Study outcomes** |
| --- | --- | --- | --- | --- | --- |
| AI-Anezi [47], Saudi Arabia | Quasi-experimental study | Cancer, diabetes, and kidney failure | 1. LLM(s): ChatGPT 3.5. 2. Scenarios and prompts: Participant inquiries. 3. Process: Participants chatted with ChatGPT as a virtual coach for chronic disease management. Participants used ChatGPT for at least 15 min daily at home, lasting 2 weeks. | 1. Methods: semistructured interviews. 2. Assessors: N.S.^b^ | Opportunities   1. Enhancing awareness of updated information on chronic disease management (e.g., diet and physical activities). 2. Reducing reliance on healthcare specialists and offering scalable support. 3. Offering free access that reduced disparities in accessing health information in chronic diseases, especially among those from rural areas. 4. Motivating health goals and promoting health behaviours regarding diets, meditation, sleep, and exercise. 5. Adapting to their preferences and needs during communication. 6. Linking to online communities to facilitate social support.   Challenges   1. Lack of physical examinations in monitoring comorbidities and potential for inaccurate diagnoses. 2. Lack of empathy for patients. 3. Ineffective in analysing complex conditions regarding chronic diseases. 4. Concerns about privacy and security for personal health data. |
| Alanezi et al. [48], Saudi Arabia | Quasi-experimental study | Chronic mental health conditions, including anxiety, depression, and behaviour disorders | 1. LLM(s): ChatGPT 3.5. 2. Scenarios and prompts: participant inquiries. 3. Process: Participants chatted with ChatGPT to seek support for managing their mental health issues for at least 15 min per day at home, lasting 2 weeks. | 1. Methods: Semistructured interviews. 2. Assessors: N.S.^b^ | Opportunities   1. Improving mental health literacy and enhancing mental health symptoms management through psychoeducation. 2. Provide a non-judgemental channel to express their concerns and offer compassionate responses. 3. Setting achievable health goals and developing plans to improve mental health. 4. Recommending mental health resources and suggesting ways to seek medical support. 5. Facilitating self-assessment and self-care practices regarding mental health symptoms. 6. Providing cognitive behaviour therapy techniques and facilitating psychotherapeutic exercises to manage negative thoughts. 7. Providing crisis intervention support for those experiencing acute distress.   Challenges   1. Potential ethical and legal concerns in data privacy, confidentiality, and biases. 2. Accuracy and reliability issues. 3. Lacking capabilities in assessing mental health conditions. 4. Not fully equipped to understand and address cultural and linguistic diversities. |
| Alanezi. [21], Saudi Arabia | Quasi-experimental study | Cancer | 1. LLM(s): ChatGPT 3.5. 2. Scenarios and prompts: Participant inquiries. 3. Process: Participants chatted with ChatGPT to seek information about cancer and its prevention and management measures, lasting 2 weeks. | 1. Methods: Focus group interviews. 2. Assessors: A researcher. | Opportunities   1. Improving health knowledge about cancer, its treatment and management of side effects without language barriers. 2. Facilitating self-management behaviours by motivating, reminding, monitoring, and tracking. 3. Connecting with social resources and support for cancer management. 4. Enhancing emotional and peer support.   Challenges   1. Privacy and reliability concerns. 2. Lack of personalisation. |
| Aliyeva et al. [35], USA^c^ | Simulation study | Severe hearing loss | 1. LLM(s): ChatGPT 4.0. 2. Scenarios and prompts: Frequently asked postoperative questions (n = 5) by patients and their families gathered by experienced otolaryngologists over three months. 3. Process: Five postoperative questions were posed to ChatGPT 4.0, focusing on symptoms after cochlear implant surgery, caring for the implant site, hearing, activities, and management after implantation. | 1. Methods: Survey to evaluate accuracy, response time, clarity, understandability, and relevance of the responses. 2. Assessors: Five specialists in otolaryngology. | Feasibility   1. Accuracy: Aligned with guidelines for cochlear implant postoperative care, ChatGPT 4.0 responses were 100% accurate. 2. Response time: Questions were answered within seconds, suggesting rapid response efficiency. 3. Clarity and understandability: The average clarity and understandability score reached 98%. 4. Relevance: The relevance of responses averaged 92%. |
| Choo et al. [46], South Korea | A simulation study | Stage IV, recurrent, synchronous colorectal cancers | 1. LLM(s): ChatGPT^TM^. 2. Scenarios and prompts: Stage IV and recurrent colorectal cancer cases discussed in the multidisciplinary tumour board at a tertiary institution, followed by inquiry treatment options. 3. Process: Patient information, including demographics and clinical history, was entered into the ChatGPT^TM^ to generate treatment recommendations. | 1. Methods: Concordance rates between ChatGPT^TM^ and the treatment recommendations made by a multidisciplinary tumour board. 2. Assessors: N.S.^b^ | Feasibility   1. Concordance rates: Approximately 73.3% of the cases were concordant with the first treatment recommendation by ChatGPT^TM^. The oncological management recommendation concordance rate between ChatGPT™ and the multidisciplinary team was 86.7%. |
| Dergaa et al. [49], Qatar | A simulation study | Mental health | 1. LLM(s): ChatGPT. 2. Scenarios and prompts: Three imaginary patient scenarios represented different mental health problems. Prompts focused on mental health and sleep management. 3. Process: ChatGPT was engaged as a virtual psychiatric provider to interact with imaginary patients following a structured format, including providing an overview of the patient’s mental health problems and detailed treatment recommendations. | 1. Methods: Qualitative assessment. 2. Assessors: academic healthcare professors. | Opportunities   1. ChatGPT provides quick responses and simulates empathy. 2. ChatGPT recommended non-pharmacological interventions as a first-line option for a simple patient scenario (a male college student), aligning with current guidelines and clinical standards of care.   Challenges   1. ChatGPT cannot interact with users and ask for further clarification. 2. Could not recommend customised assessment and treatment plans for complex patient scenarios (e.g., a female with systemic lupus erythematosus). |
| Dergaa et al. [50], Qatar | A simulation study | Arterial hypertension, osteoarthritis, anxiety and stress-related issues, diabetes, asthma and reduced pulmonary function | 1. LLM(s): ChatGPT 4.0. 2. Scenarios and prompts: Five hypothetical patient profiles with different health conditions focusing on cardiovascular health, musculoskeletal strength, mental health, diabetes, and respiratory health management, respectively. Prompts focused on generating a 30-day fitness program table based on the scenarios. 3. Process: Five scenarios with patient profiles (e.g., sex, age, height, basal metabolic rate, and medication intake) were created to test the ability of ChatGPT 4.0 to prescribe a 30-day fitness program. | 1. Methods: qualitative assessment. 2. Assessors: a panel of academic professors and doctors in exercise science or medicine. | Feasibility   1. ChatGPT 4.0 could create general safety-conscious exercise programmes, which adhered to FITT^d^ (Frequency, intensity, time, and type) principles, RPE^e^ (rate of perceived exertion) guidelines, and research evidence.   Challenges   1. Prescribed plans did not vary. ChatGPT 4.0 prioritised excessive safety over effectiveness of training and might fall short of providing necessary stimuli for health improvement. 2. ChatGPT 4.0 lacked preliminary patient assessment and was unable to monitor physiological response and adjust personalised physical exercise regimens in real-time. |
| Franco D’Souza et al. [51], India | A simulation study | Psychiatric disorders | 1. LLM(s): ChatGPT 3.5. 2. Scenarios and prompts: 100 clinical case vignettes from a book representing different psychiatric illnesses, which were used to converse with ChatGPT 3.5 to generate responses. | 1. Methods: Grading. 2. Assessors: two experts with clinical experience in psychiatry. | Feasibility   1. By assessing coverage of a standard answer, the responses of ChatGPT 3.5 in 61, 31, and 8 of 100 cases received ‘Grade A’, ‘Grade B’, and ‘Grade C’ ratings, respectively. 2. ChatGPT performed well in generating management strategies followed by diagnosis for psychiatric conditions. |
| Kianian et al. [36], USA^c^ | A simulation study | Glaucoma | 1. LLM(s): ChatGPT. 2. Scenarios and prompts: Seven prompts were given to ChatGPT to generate patient handouts regarding surgery management of glaucoma with simple language and to include references. | 1. Methods: readability was assessed using FKRE^f^, FKGL^g^, GFI^h^, and SMOG^i^. 2. Assessors: two authors. | Feasibility   1. Readability: The health information generated by ChatGPT was easily readable compared to webpages (9th-grade reading level vs. 11th-grade reading level). Readability did not differ significantly. 2. Quality: ChatGPT scored the quality of health resources with high precision (r = 0.725, *P* < 0.001). |
| Lim et al. [45] Singapore | A simulation study | Colorectal cancer | 1. LLM(s): Contextualised ChatGPT 4.0. The ChatGPT 4.0 model was contextualised based on guidelines for colorectal cancer screening and surveillance, which were processed into a knowledge base. The process included splitting each article into textual chunks and embedding each chunk based on similarities between words to allow fast searching by the model. 2. Scenarios and prompts: 62 hypothetical patient scenarios with or without risks of colorectal cancer. 3. Process: The contextualised ChatGPT 4.0 model was instructed to recommend colonoscopy screening intervals in each simulated patient scenario. | 1. Methods: scoring approach (accuracy) and content comparison (hallucination). 2. Assessors: three gastroenterology fellows under the supervision of two senior gastroenterologists. | Feasibility   1. Accuracy: Compared with the standard ChatGPT 4.0 model, the contextualised model performed better in recommending correct screening intervals overall (79% vs. 50.5%, *P* < 0.01) and in each patient risk category, including screening (87.7% vs. 52.6%, p <0.01), surveillance (63.2% vs. 40.2%, *P* < 0.01), post-cancer surveillance (100% vs. 67.7%), and others (100% vs. 70.8%).   Challenges   1. The contextualised ChatGPT 4·0 failed to identify a high-risk feature in one response and experienced hallucination in two responses. By contrast, the standard ChatGPT 4.0 failed to identify high-risk features in 12 responses and hallucinated a high-risk feature in 13 responses. |
| Mondal et al. [52], India | A simulation study | A set of 20 lifestyle-related chronic diseases, including obesity, diabetes, cardiovascular health, and mental health | 1. LLM(s): ChatGPT 3.5. 2. Scenarios and prompts: 20 cases mimicking individuals seeking information related to lifestyle-related diseases. 3. Process: 20 cases were formulated into four questions and presented to ChatGPT for generating relevant answers. | 1. Methods: Readability was assessed using the FKRE^f^ and FKGL^g^. Qualitative ratings were used to assess the accuracy and applicability of responses. 2. Assessors: two academic primary care physicians. | Feasibility   1. Readability: The mean FKRE^f^ score was 27.8 (5.74), suggesting the generated text was understandable by college students. 2. Accuracy and applicability: The average accuracy and applicability scores of the ChatGPT responses were 1.83 and 1.9, respectively, significantly higher than the hypothesized median score of 1.5. |
| Papastratis et al. [53], Greece | A simulation study | Noncommunicable diseases (e.g., obesity, diabetes, and cardiovascular diseases) | 1. LLM(s): ChatGPT 3.5 and ChatGPT 4. 2. Scenarios and prompts: 15 user profiles (e.g., physical characteristics and medical conditions) for patients with noncommunicable diseases, including obese adults and adults with cardiovascular diseases. 3. Process: To generate weekly meal plans per day for patients with non-communicable diseases by interacting with their profiles, including body mass index, basal metabolic rate, and types of non-communicable diseases. Information including meal types (including breakfast, morning snack, lunch, afternoon snack, dinner, and supper), calories, total protein, total fat, and total number of vegetables were outputted. | 1. Methods: Accuracy and variability of the meal plans were assessed based on guidelines and unique numbers of meals. 2. Assessors: N.S.^b^ | Feasibility   1. Accuracy: Compared with a knowledge-based recommender (91%), ChatGPT 3.5 (81.53%) and ChatGPT 4.0 (81.62%) had lower nutrient accuracy rate for users with non-communicable diseases overall. By inputting personalised target energy intake, the nutrient accuracy rate was improved to 86% in ChatGPT 4.0; and the average caloric difference was 17% and 3% in ChatGPT 3.5 and ChatGPT 4.0, suggesting that the recommended energy intake was comparable to the user’s suggested energy intake. 2. Variability: The average meal variety was highest in ChatGPT 3.4 (6.58), followed by ChatGPT 4.0 (6.40), and the knowledge-based recommender (4.89). |
| Pradhan et al. [37], USA^c^ | A simulation study | Liver cirrhosis | 1. LLM(s): ChatGPT 4.0, DocsGPT, Google Bard, and Bing Chat. 2. Scenarios and prompts: direct inquiries for generating patient educational materials on cirrhosis. 3. Process: 1-page patient education sheet instructing patients about cirrhosis. | 1. Methods: Readability and grade level were assessed using FKRE^f^, SMOG^i^, and FKGL^g^. 2. Assessors: 14 patients/caregivers and 8 transplant hepatologists. | Feasibility   1. Readability: Compared with human-derived health educational materials, LLM-generated materials had higher FKRE^f^ scores in, indicating they were easier to comprehend. The FKGL^g^ ranged from 5.5 to 7.9 in LLM-generated materials, indicating they were comprehendible by people with an eighth-grade educational level. Based on the SMOG^i^, LLM-generated materials were expected to be comprehensible by people with high school educational levels or above (ranging from 9.4 to 12.3), except that in Google Bard-derived educational material. 2. Actionability: No significant difference in the scores for actionability between the human-derived and LLM-derived educational materials (p > 0.05). Only human-derived health educational material met the actionable score cut-off of ≥70%. 3. Accuracy: The health educational materials generated by humans and LLMs (Bing Chat, ChatGPT 4.0, and Google Bard) were considered to contain 76% to 99% accurate information, as assessed by hepatologists. |
| Puerto Nino et al. [43], Canada | A simulation study | Benign prostate enlargement | 1. LLM(s): ChatGPT 4.0+. 2. Scenarios and prompts: 88 benign prostate enlargement-centric queries were formulated based on the patient frequently asked questions form on the European Association of Urology and American Urological Association websites. 3. Process: 88 queries related to symptoms, diagnoses, complications, and treatment options of benign prostate enlargement were fed to ChatGPT independently to generate responses. | 1. Methods: Performance metrics and general quality score using a 5-point Likert scale. 2. Assessors: Two examiners. | Feasibility   1. Performance: Precision score ranged from 0.50 to 1, with an overall performance score of 0.66. Recall score ranged from 0.9 to 1.0, with an overall performance score of 0.97. 2. General quality score: A median general quality score of 4 was obtained. |
| Seth et al. [41] Australia | A simulation study | Carpal tunnel syndrome | 1. LLM(s): ChatGPT (no version number). 2. Scenarios and prompts: six questions regarding the diagnosis and management of carpal tunnel syndrome. 3. Process: six inquiries with common clinical scenarios of carpal tunnel syndrome were inputted into ChatGPT to generate management strategies. | 1. Methods: Efficacy and performance of ChatGPT were assessed using a 5-point Likert scale. 2. Assessors: Two experienced plastic surgeons. | Feasibility   1. Accuracy: ChatGPT recommended concise treatment options and considered personal factors in recommending treatment choices for carpal tunnel syndrome. ChatGPT accurately evaluated the efficacy of surgical and nonsurgical treatments for carpal tunnel syndrome in short- and long-term outcomes. ChatGPT accurately identified the diagnosis and recommended further investigation (e.g., physical examination and nerve conduction) and management strategies in an easy-to-understand manner. ChatGPT correctly identified the deterioration of the symptoms and recommended seeking appropriate medical attention.   Challenges   1. There were erroneous references and only three of the five references could be found in the literature. 2. Although it retrieved level I evidence, the depth and detail of the information (e.g., statistics of recurrence rates) were not sufficient. 3. ChatGPT cited two seminal and three nonexistent ‘recent’ studies to support its answer. |
| Singer et al. [38] USA^c^ | A simulation study | Eye care | 1. LLM(s): *Aeyeconsult* powered by ChatGPT 4·0. 2. Scenarios and prompts: 260 questions from OphthoQuestions.com. 3. Process: Based on textbook source material, *Aeyeconsult* was developed by integrating Lang Chian to extract and split texts of source materials and user queries into chunks with unique values and stored into a vector store with Pinecone. Via comparison with chunks of user queries, the 10 most similar chunks of texts were identified to serve as context to generate a natural language response. Only the first response was recorded as an answer. | 1. Methods: The correct rates of the answers given by ChatGPT 4·0. 2. Assessors: N.S.^b^ | Feasibility   1. Rates of correct responses: *Aeyeconsult* performed more accurately than ChatGPT 4.0 (83.4% vs. 69.2%, p = 0.0118) regarding the 260 questions from OphthoQuestions. ChatGPT 4.0 performed worst in Retina and Vitreous (37.5%). *Aeyeconsult* had the lowest accuracy rate (68.1%) in clinical optics than other categories (e.g., cornea, fundamentals, general medicine, glaucoma, and paediatrics), whereas it still outperformed ChatGPT 4.0 (45.5%) in this category. 2. No answers*: Aeyeconsult* had fewer no answers than ChatGPT 4.0 (5 vs. 18). 3. Multiple answers*: Aeyeconsul*t had fewer multiple answers than ChatGPT 4.0 (0 vs. 7). 4. Consistency of answers: *Aeyeconsult* had complete consistency for questions initially answered correctly. In contrast, ChatGPT gave different responses to 3 of the 13 questions initially answered correctly. For questions initially answered incorrectly (n = 13), *Aeyeconsult* provided different answers for 8 of the questions (61.5%) and ChatGPT 4.0 provided different responses for all of the 13 questions (100%) over 10 attempts. |
| Spallek et al. [42], Australia | A simulation study | Mental health and substance use disorders | 1. LLM(s): ChatGPT 4.0 pro. 2. Scenarios and prompts: direct user queries and four factsheets related to mental health and substance use were chosen from educational portals. 3. Process: queries from Positive Choices and Cracks in the Ice were used to prompt ChatGPT 4.0 to capture real-world communication. | 1. Methods: readability (using Sydney Health Literacy Lab), quality, and following guidelines were assessed. 2. Assessors: Study authors. | Feasibility   1. Readability: The ChatGPT 4·0 outputs generated a desirable low text complexity rating, ranging from 24% to 33%, indicating fewer uncommon words, medical jargon, and acronyms. The reading levels were higher in ChatGPT 4·0-generated materials using direct user queries (grade 13.9, SD^j^ 1.52) and two types of engineered prompts (grade 13.1, SD^j^ 3.20; grade 12.9, SD^j^ 1.20) than that in expert-developed factsheets (grade 12.2, SD^j^ 1.44). 2. Adherence to communication guidelines: ChatGPT 4.0 responses to direct user queries and simple prompts had lower average adherence to communication guidelines, with 23% (5/22) of the outputs having at least one stigmatising phrase. ChatGPT 4.0 outputs responding to engineered prompts were more likely to have a cautionary tone and disclaimers than direct queries and simple prompts. 3. Quality of advice: ChatGPT 4.0 outputs featured a high level of accuracy without hallucinations and could tailor target audiences, whereas it lacks breadth and depth of expertise compared to human experts. |
| Willms & Liu [44], Canada | An autoethnographic case study | Disease prevention by increasing physical activity | 1. LLM(s): ChatGPT 3.0 combined with Pathverse, a no-code app. 2. Scenarios and prompts: prompts were developed based on topics including parental support, active attitudes, and self-monitoring of physical activity. The prompts also considered instruction, context information, and output indicators (e.g., word count). Generated contents were added to Pathverse. 3. Process: Based on the multi-process action control framework, ChatGPT 3.0 was used to generate just-in-time adaptive interventions addressing the intention-behaviour gap to support parents in helping their children (8–12 years old) be physically active. | 1. Methods: Acceptability, relevance, and tone of responses were assessed based on field notes and discussions. Future recommendations were also suggested. 2. Assessors: two researchers. | Feasibility   1. Accuracy and relevance: ChatGPT had acceptable accuracy and relevance in responding to prompts, whereas it might provide false academic references. 2. Tone of responses: acceptable for research purposes and matched the prompts given (in a fun and positive voice). |
| Yang et al. [39], USA^c^ | A case study | Diet management for preventing chronic illnesses | 1. LLM(s): *ChatDie*t based on ChatGPT 3.5 Turbo. 2. Scenarios and prompts: users’ inquiries and individual-specific information, including personal food preferences, dietary history, health records, and physiological signals gathered from wearable devices. Synthetic participants (n = 100). 3. Process: *ChatDiet* included Orchestrator to interact with personal (e.g., causal discovery) and population models (e.g., food nutrition list loading) to extract information related to diet based on users’ inquiries. The aggregated information was sent to ChatGPT 3.5 Turbo to integrate with its internal knowledge and realise its interactions with the users. The tasks involved retrieving (filtering and retrieving relevant information based on users’ inquiries from the individual and population models), transcribing (converting data into textual information), and promoting engineering to instruct ChatGPT 3.5 to provide food recommendations adhering to inputs. | 1. Methods: Quantitative causal graphs and qualitative analyses of the outputs were implemented. 2. Assessors: researchers. | Feasibility   1. Effectiveness: The recommendation effectiveness ratio of personalised food recommendations ranged from 85% to 95% for heart rate variability, sleep quality, and duration. 2. Personalisation: *ChatDiets* can adapt to personal needs by considering an individual’s unique nutritional nuances based on personal nutrition effects. 3. Interactivity: *ChatDiets* demonstrated interactivity within the recommendation process by initiating follow-up questions and offering alternative options when users lacked interest in the suggested foods.   Challenges   1. Food suggestions were confined to the factors in the dataset. Nonsensical recommendations and hallucinations occurred in some cases. |
| Yeo et al. [40], USA^c^ | A simulation study | Liver cirrhosis and hepatocellular carcinoma | 1. LLM(s): ChatGPT Dec 15 version. 2. Scenarios and prompts: 73 questions and 91 questions were selected for hepatocellular carcinoma and cirrhosis posted by well-recognised professional societies and institutions and posts by patient support groups on Facebook. 3. Process: 164 questions were entered into ChatGPT twice, and both responses were recorded and assessed. | 1. Methods: Grading and qualitative assessment. 2. Assessors: two transplant hepatologist reviewers. | Feasibility   1. Accuracy: ChatGPT had 79.1% and 74.0% accurate rate in knowledge of cirrhosis and hepatocellular carcinoma. 76.9% accuracy rate (20/26 quality measures) in the knowledge regarding cirrhosis management. 50% accuracy rate in knowledge of hepatocellular carcinoma screening, compared to 28.8% to 45.4% responded by physicians.   Opportunities   1. Acknowledged potential emotional responses and provided actional suggestions and motivational responses to treating and managing hepatocellular carcinoma. 2. Psychological and practical emotional support given to patient caregivers.   Challenges   1. Failed to identify correct cut-offs for and window time for specific conditions (e.g., liver transplantation). 2. Failed to identify age cut-off, screening tests, and surveillance eligibility of hepatocellular carcinoma. |

^a^LLM: large language model

^b^N.S.: not specified

^c^USA: the United States of America

^d^FITT: Frequency, intensity, time, and type

^e^RPE: rate of perceived exertion

^f^FKRE: Flesch-Kincaid reading ease score

^g^FKGL: Flesch–Kincaid Grade Level

^h^GFI: Gunning Fog index

^i^SMOG: simple measure of gobbledygook

^j^SD, standard deviation
