# Supplementary material for: Unveiling the Potential of Large Language Models in Transforming Chronic Disease Management: Mixed Methods Systematic Review
Source: J Med Internet Res. 2025 Apr 16;27:e70535. doi: 10.2196/70535 (PMC12044321; doi:10.2196/70535)
Supplement: Multimedia Appendix 4 [file jmir_v27i1e70535_app4.docx]

**
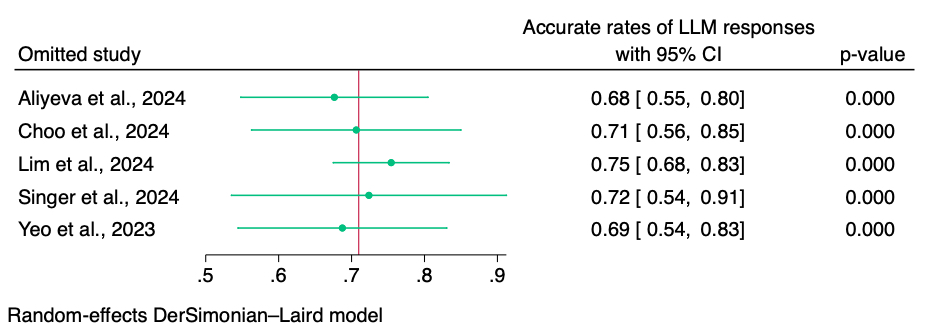
**

**Figure S1. Sensitivity analysis for accurate rates of LLM responses.**

**Table S1** Appraisal of the methodological quality of included simulation and case studies using a rating rubric (n = 17)

| **Appraisal items** | Alanezi. [21], 2024 | Choo et al. [46], 2024 | Dergaa et al. [49], 2024 | Dergaa et al. [50], 2024 | Franco D’Souza et al. [51], 2023 | Kianian et al. [36], 2024 | Lim et al. [45], 2024 | Mondal et al. [52], 2023 | Papastratis et al. [53], 2024 |
| --- | --- | --- | --- | --- | --- | --- | --- | --- | --- |
| Introduction/Background/Rationale | 4 | 4 | 4 | 4 | 4 | 4 | 4 | 3 | 4 |
| Literature review | 2 | 2 | 3 | 4 | 4 | 3 | 4 | 4 | 4 |
| Problem Statement/Objective of study/Research questions | 4 | 4 | 4 | 4 | 4 | 4 | 4 | 4 | 4 |
| Guiding conceptual or theoretical framework | 0 | 0 | 0 | 0 | 0 | 0 | 0 | 0 | 0 |
| Study design | 3 | 4 | 4 | 4 | 4 | 4 | 4 | 4 | 4 |
| Strengths of study design:  Quantitative | 3 | 3 | NA | NA | 3 | 4 | 4 | 3 | 4 |
| Strengths of study design:  Qualitative | NA | NA | 2 | 2 | NA | NA | NA | NA | NA |
| Sample and setting | 1 | 2 | 1 | 1 | 3 | 2 | 3 | 2 | 2 |
| Simulation development | 3 | 4 | 4 | 4 | 3 | 4 | 4 | 4 | 4 |
| Description of simulation implementation | 3 | 4 | 4 | 4 | 4 | 4 | 4 | 4 | 4 |
| Description of simulation feedback or debriefing | NA | NA | NA | NA | NA | NA | NA | NA | NA |
| Study instruments:  Quantitative studies | 2 | 3 | NA | NA | 2 | 4 | 4 | 4 | 2 |
| Study instruments:  Qualitative studies | NA | NA | 2 | 2 | NA | NA | NA | NA | NA |
| Results | 4 | 4 | 2 | 2 | 4 | 4 | 4 | 4 | 4 |
| Discussion | 3 | 4 | 4 | 4 | 4 | 4 | 4 | 4 | 1 |
| IRB or ethics committee approval/exemption | NA | 4 | NA | NA | NA | NA | NA | NA | NA |
| **Total score** | **32/48=66.7%** | **42/52=80.8%** | **34/48=70.8%** | **35/48=72.9%** | **39/48=81.3%** | **41/48=85.4%** | **43/48=89.6%** | **40/48=83.3%** | **37/48=77.1%** |

**Table S1** Appraisal of the methodological quality of included simulation and case studies using a rating rubric (n = 17, continued)

| **Appraisal items** | Pradhan et al. [37], 2024 | Puerto Nino et al. [43], 2024 | Seth et al. [41], 2023 | Singer et al. [38], 2024 | Spallek et al. [42], 2023 | Willms and Liu [44], 2024 | Yang et al. [39], 2024 | Yeo et al. [40], 2023 |
| --- | --- | --- | --- | --- | --- | --- | --- | --- |
| Introduction/Background/Rationale | 2 | 4 | 2 | 3 | 4 | 4 | 4 | 4 |
| Literature review | 2 | 4 | 2 | 2 | 4 | 4 | 4 | 4 |
| Problem Statement/Objective of study/Research questions | 4 | 4 | 4 | 4 | 4 | 4 | 4 | 4 |
| Guiding conceptual or theoretical framework | 0 | 0 | 0 | 0 | 0 | 4 | 0 | 0 |
| Study design | 4 | 4 | 4 | 4 | 4 | 4 | 4 | 3 |
| Strengths of study design:  Quantitative | 4 | 3 | NA | 4 | 3 | NA | 4 | 4 |
| Strengths of study design:  Qualitative | NA | NA | 2 | NA | NA | 3 | NA | NA |
| Sample and setting | 1 | 3 | 2 | 4 | 2 | 1 | 3 | 3 |
| Simulation development | 3 | 4 | 4 | 4 | 4 | 4 | 4 | 4 |
| Description of simulation implementation | 4 | 4 | 4 | 4 | 4 | 4 | 4 | 4 |
| Description of simulation feedback or debriefing | NA | NA | NA | NA | NA | NA | NA | NA |
| Study instruments:  Quantitative studies | 4 | 3 | NA | 3 | 2 | NA | 2 | 3 |
| Study instruments:  Qualitative studies | NA | NA | 1 | NA | NA | 1 | NA | NA |
| Results | 4 | 4 | 4 | 4 | 4 | 4 | 4 | 4 |
| Discussion | 4 | 4 | 4 | 4 | 2 | 4 | 3 | 4 |
| IRB or ethics committee approval/exemption | NA | NA | NA | NA | NA | NA | NA | NA |
| **Total score** | **36/48=75%** | **41/48=85·4%** | **33/48=68.8%** | **40/48=83.3%** | **37/48=77.1%** | **41/48=85.4%** | **40/48=88.3%** | **41/48=85.4%** |

**Table S2** Appraisal of the methodological quality of included quasi-experimental study using ROBINS-I (n = 3)

| Study | Bias due to confounding | Bis due to selection of participants | Bias in classification of interventions | Bias due to deviations from intended interventions | Bias due to missing data | Bias in measurement of outcomes | Bias in selection of the reported result | Overall |
| --- | --- | --- | --- | --- | --- | --- | --- | --- |
| AI-Anezi [47], 2024 | Moderate | Low | Moderate | Moderate | Low | Serious | Low | Serious |
| Alanezi et al. [48], 2024 | Moderate | Low | Moderate | Moderate | Low | Serious | Low | Serious |
| Alanezi. [21], 2024 | Moderate | Low | Moderate | Moderate | Low | Serious | Low | Serious |
